# Efficacy and Safety of Mepolizumab in Patients With Eosinophilic Granulomatosis With Polyangiitis: A Single-Center Experience

**DOI:** 10.7759/cureus.68282

**Published:** 2024-08-31

**Authors:** Mohamed Abuzakouk, Said Isse, Mike Wechsler, Mateen Uzbeck, Rajaie A Namas, Omar Ghorab, Asaad Idris, Ali Wahla, Hamad Alhameli, Fulvio Salvo, Zaid Zoumot, Irfan Shafiq

**Affiliations:** 1 Respiratory and Allergy Institute, Cleveland Clinic Abu Dhabi, Abu Dhabi, ARE; 2 Pulmonary and Critical Care Medicine, National Jewish Health, Denver, USA; 3 Rheumatology, Cleveland Clinic Abu Dhabi, Abu Dhabi, ARE

**Keywords:** biologics, vasculitis, asthma, eosinophilic granulomatosis with polyangiitis, mepolizumab

## Abstract

Eosinophilic granulomatosis with polyangiitis (EGPA), previously known as Churg-Strauss syndrome, is a systemic vasculitis characterized by eosinophil-rich, necrotizing granulomatous inflammation primarily affecting the respiratory tract with necrotizing vasculitis of small- to medium-sized arteries. In this case series, we retrospectively evaluated the efficacy and safety of mepolizumab in seven patients diagnosed with EGPA who presented to the Department of Allergy and Clinical Immunology at Cleveland Clinic Abu Dhabi. The variables assessed before and after mepolizumab treatment included Birmingham Vasculitis Activity Score (BVAS), prednisolone dose, Asthma Control Test (ACT) score, and blood eosinophil count (BEC). We found a significant reduction in BVAS and prednisolone dosage with clinical improvements in asthma symptoms after treatment with mepolizumab. Our case series, the first from the Middle East on the use of mepolizumab in EGPA, demonstrates that mepolizumab is a safe and effective treatment for patients with EGPA.

## Introduction

Eosinophilic granulomatosis with polyangiitis (EGPA) is an uncommon vasculitis disorder affecting multiple organ systems and is marked by asthma, lung infiltrates, sinusitis, neuropathy, and increased peripheral eosinophils. It also impacts the heart, skin, kidneys, and gastrointestinal tract [1,2]. Eosinophils are thought to play a central role in the pathogenesis of EGPA with previous studies suggesting that eosinophils infiltrate tissue and vasculature, releasing inflammatory mediators that lead to inflammation, especially the highly cationic eosinophil granule proteins, which damage tissue by producing highly reactive free oxygen radicals and cause clinical manifestations [3]. Furthermore, ANCA-mediated activation of neutrophils leads to endothelial damage through the release of cytotoxic enzymes and priming of alternative complement pathways to release inflammatory cytokines leading to a vicious cycle of inflammation and vasculitic process [4,5].

Standard treatment of EGPA includes early initiation of systemic glucocorticoids, often paired with an additional immunosuppressive agent (e.g., cyclophosphamide) in patients with advanced or life-threatening disease [6]. Once remission is achieved, glucocorticoids are tapered, and cyclophosphamide is replaced with a less toxic immunosuppressant such as azathioprine or methotrexate. However, one study suggested that the addition of azathioprine as steroid-sparing therapy did not affect remission rates and was therefore ineffective in non-severe systemic necrotizing vasculitides [7]. Rituximab, an anti-CD20 monoclonal antibody, has also been used for the treatment of EGPA and other vasculitides, both for the induction of remission and treatment of relapses in a few recent studies [8-10]. Glucocorticoids work by inducing apoptosis and inhibiting pro-survival signaling pathways, thus reducing blood and tissue eosinophilia [11]. However, many patients continue to experience relapses despite treatment. In a study of 118 EGPA patients treated with glucocorticoids, disease relapse occurred in 38% of patients after treatment [12,13]. In addition, glucocorticoids are associated with significant side effects with both short- and long-term use [14,15]. Tapering of corticosteroids can also result in relapse and cause significant morbidity [16].

Mepolizumab is an anti-interleukin-5 (anti-IL-5) monoclonal antibody that reduces relapse rates and glucocorticoid use in EGPA [17,18]. Several recent studies have shown it to be effective and well tolerated, resulting in Federal Drug Administration (FDA) approval of this drug for EGPA in 2017 [19-22]. In this case series, we report the real-life experience with mepolizumab in EGPA patients attending a tertiary healthcare facility in the Middle East.

## Materials and methods

Seven patients, above 20 years of age, with EGPA attended our hospital from 2017 to 2021. All patients received several courses of oral prednisolone in addition to their regular treatment of inhaled corticosteroid/long-acting beta-agonist and had had multiple hospital admissions for severe asthma symptoms.

In all, the diagnosis was established in accordance with the American College of Rheumatology classification criteria for EGPA including the presence of at least four of the following: asthma, eosinophilia > 10%, sinusitis, pulmonary infiltrates, mononeuropathy/polyneuropathy, and presence of extravascular vasculitis. Baseline demographics, blood eosinophil count (BEC), C-reactive protein (CRP), Birmingham Vasculitis Activity Score (BVAS), Asthma Control Test (ACT), and oral corticosteroid (OCS) dosage were reviewed and recorded for all patients. Data were extracted and independently verified by two members of the research team. All immunologic blood investigations including antinuclear antibody (ANA), antineutrophil cytoplasmic antibody (ANCA), IgA, IgM, IgG, serum protein electrophoresis, urine immunofixation, C3, C4, rheumatoid factor, and anti-cyclic citrullinated peptide antibody (anti-CCP) were reviewed by the clinical immunologist.

Disease severity at baseline and after mepolizumab therapy was measured using the BVAS. Remission was defined as having a BVAS score of zero with an OCS dose of 7.5 mg or less, while relapse was defined as a BVAS score of one or more, plus one of the following: active asthma symptoms with worsening of Asthma Control Test (ACT < 20), initiation or increase of immunosuppressive therapy, or hospitalization due to EGPA-related symptoms during the study period.

Four of the seven patients were started on mepolizumab to help taper daily OCS, and the other three received mepolizumab to treat EGPA refractory to steroid therapy. All patients were initiated on mepolizumab 300 mg subcutaneously every four weeks. However, three patients later needed to receive the treatment every three weeks due to inadequate response to the four-week regimen. Patients were treated for a median of 17 months. All patients were reassessed 8-12 weeks after the initiation of mepolizumab.

The study was part of our asthma registry project and was approved by the local research ethics committee.

Statistical analysis

Due to the small number of cases included, the data are expressed as median and range. Proportion was used as descriptive statistics for categorical variables. Pre- and post-treatment analysis was done using a paired t-test, and a significant p-value was taken to be less than 0.05. Statistical analysis was performed using Statistical Package for the Social Sciences (SPSS) version 27 (IBM SPSS Statistics, Armonk, NY) and GraphPad software for Windows (GraphPad, La Jolla, CA).

## Results

The median age of the patients was 46 (60-27) years. Five of the seven subjects were female (71.4%). The median weight was 75 (57-93) kg, while the median body mass index (BMI) was 26 (23-39) kg/m^2^. The median number of mepolizumab doses was 17 (4-48) doses. Only two patients tested positive for ANCA, specifically anti-MPO p-ANCA. Urine analysis, renal and liver profiles, ANA, rheumatoid factor, anti-CCP, immunoglobulins, and serum protein electrophoresis did not show any significant abnormality. Microbiological investigations including hepatitis B and C, HIV-1 and HIV-2 serologies, and blood cultures were all negative. Patients' pre- and post-treatment parameters and patients' characteristics are summarized in Table 1 and Table 2, respectively.

**Table 1:** Patients' characteristics pre- and post-mepolizumab therapy *Relapse without hospitalization **Relapse with hospitalization ANCA: antineutrophil cytoplasmic antibody, CRP: C-reactive protein, ACT: Asthma Control Test, BVAS: Birmingham Vasculitis Activity Score

	Patient 1*	Patient 2	Patient 3	Patient 4	Patient 5**	Patient 6**	Patient 7
Age (years)	43	42	53	46	59	45	26
Gender	Female	Male	Female	Female	Male	Female	Female
Beginning of treatment	25/02/2019	10/10/2018	31/05/2020	13/10/2020	14/10/2020	26/04/2017	01/03/2020
ANCA status	Negative	Negative	Negative	Positive	Negative	Positive	Negative
Pre-mepolizumab eosinophil count (10^9^/L)	0.06	0.8	0.15	0	0.04	0.08	0.01
Post-mepolizumab eosinophil count (10^9^/L)	0.04	0.07	0.04	-	0.03	-	0.044
Pre-mepolizumab CRP (mg/L)	5.7	3.3	10.8	1	1.8	93.62	54.5
Post-mepolizumab CRP (mg/L)	8.4	6.2	1.3	0.6	-	14.8	-
Pre-mepolizumab ACT score	20	21	-	19	-	12	-
Post-mepolizumab ACT score	14	25	25	24	-	25	22.6
BVAS (pre)	1	1	0	2	3	0	1
BVAS (post)	0	0	0	0	0	0	0
Pre-mepolizumab prednisolone	10	5	10	40	30	12.5	25
Post-mepolizumab prednisolone	0	0	2.5	2.5	2.5	0	0

**Table 2:** Baseline characteristics ANCA: antineutrophil cytoplasmic antibody

	Number (%)	Median (range)
Gender		
Male	2 (28.6%)	
Female	5 (71.4%)	
Age		46 (60-27)
Weight (kg)		75 (93-57)
Number of doses		17 (48-4)
Disease relapse	3 (43%)	
ANCA positive	2 (29%)	

Response to mepolizumab in terms of differences in BEC, CRP, ACT, BVAS, and OCS dose is shown in Table 3.

**Table 3:** Response to mepolizumab treatment Results are expressed as median (range), and p-values are for the paired t-test. CRP: C-reactive protein, ACT: Asthma Control Test, BVAS: Birmingham Vasculitis Activity Score, OCS: oral corticosteroids

	Pre-treatment	Post-treatment	p-value
Eosinophils count	0.06 (0.8-0)	0.04 (0.07-0.03)	0.28
CRP	5.7 (93.6-1)	6.2 (14.8-0.6)	0.35
ACT	19 (21-12)	23 (25-14)	0.38
BVAS	1 (3-0)	0 (0)	0.03
OCS	12.5 (40-0)	2.5 (10-0)	0.04

**Figure 1:**
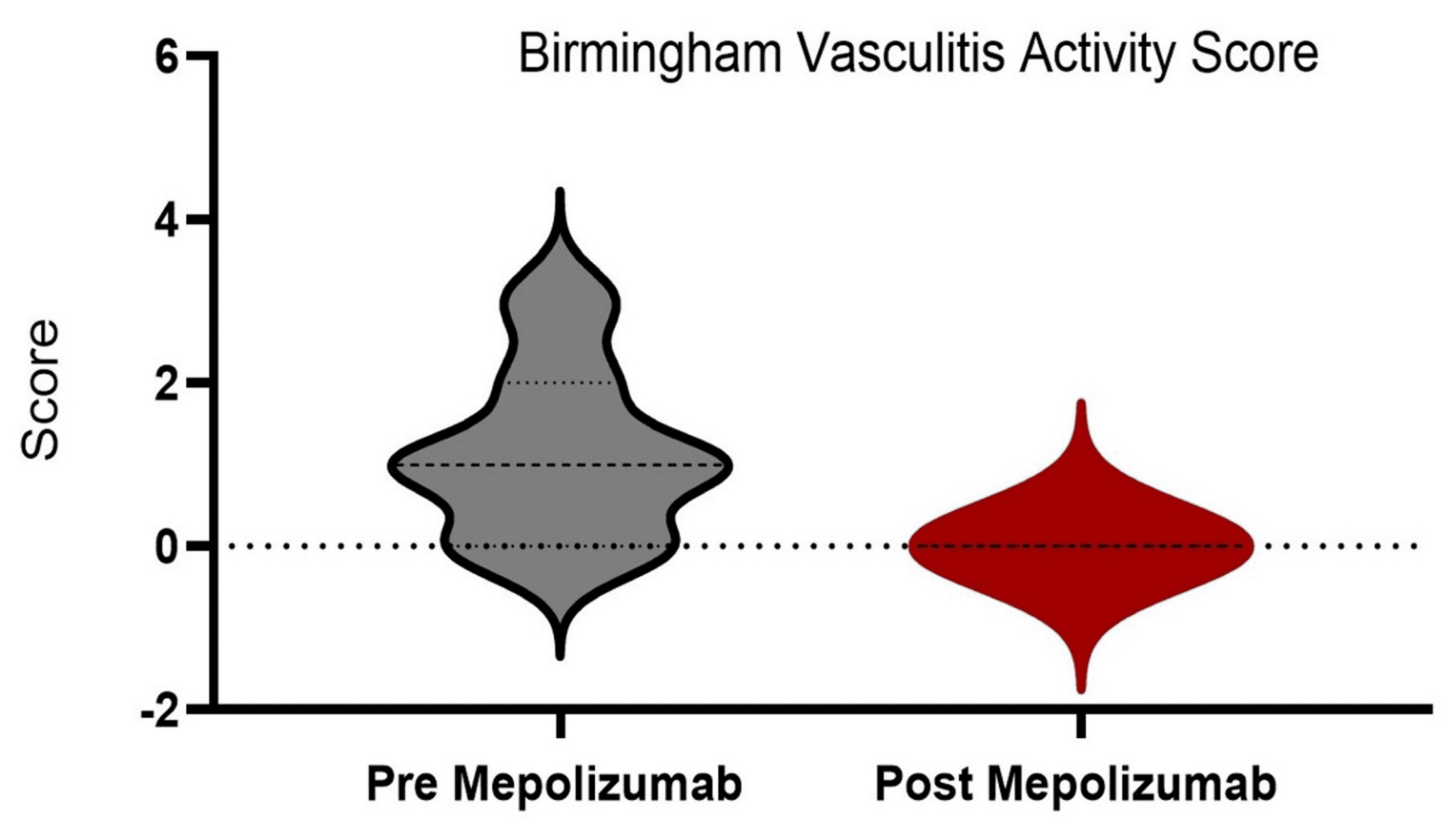
Response to mepolizumab using BVAS as an assessment tool BVAS: Birmingham Vasculitis Activity Score

Participants demonstrated a significant reduction in BVAS from one unit to zero (p=0.03) and corticosteroid dosage from 12.5 to 2.5 (p=0.04) (Figure 1 ).

The adjusted linear regression model was used to assess patient factors influencing changes in BVAS and OCS dosage; however, no factor showed a significant effect, and the change was independent (Table 4).

**Table 4:** Patient factors impacting the response to mepolizumab treatment BVAS: Birmingham Vasculitis Activity Score

Factors	Estimate (p-value)	
	Change in BVAS	Change in dosage
Age	0.006 (0.87)	0.3 (0.54)
Gender	0.04 (0.96)	-19 (0.25)
Weight	-1.19 (0.25)	-2 (0.15)
Number of mepolizumab doses	0.1 (0.13)	2 (0.09)

All patients achieved remission after mepolizumab therapy; the median number of doses required to achieve remission was 4 (7-3). After achieving remission, four (57%) patients did not experience any relapses for a median of 10 (14-6) months. Three patients relapsed, with two of them necessitating hospitalization. All three had severe exacerbation of asthma, requiring systemic corticosteroids, but not antibiotics. Two patients who were hospitalized experienced arthralgia as well.

At the end of the study period (June 2021), all patients had a BVAS of zero with optimum control of their EGPA symptoms including asthma (ACT > 20).

Adverse events and discontinuation

No serious adverse events were recorded during the study period.

## Discussion

In this retrospective real-world analysis, we demonstrate that mepolizumab is an effective and safe therapy for relapsing EGPA. All seven patients achieved remission after a median of four months, with over half of the patients remaining in remission for up to 10 months of treatment. Moreover, all patients had a BVAS of zero with optimum asthma control (ACT > 20) by the end of the study period. Similar results were reported by Wechsler et al., who showed that mepolizumab led to significantly more accrued weeks of remission compared to placebo [17]. Most recently, in a multicenter observational study, Nolasco et al. showed mepolizumab to be effective in inducing remission in 23 patients with EGPA. They also demonstrated a reduction in eosinophil count, daily corticosteroid dose, and BVAS along with improved lung function [23].

We also showed a statistically significant improvement in BVAS (p=0.03) with mepolizumab therapy, albeit in a very small patient group. Previously, Ueno et al. also reported improvements in BVAS after 12 months of mepolizumab therapy in a 16-patient series with refractory EGPA; however, in their cohort, only 75% of patients achieved remission within 12 months of starting treatment [22].

The high burden of OCS treatment is a major concern for patients with refractory and relapsing EGPA. In our cohort, we found a statistically significant reduction in daily OCS use (12.5 mg to 2.5 mg, p<0.05) after treatment with mepolizumab. Moreover, more than 50% of patients who were on daily OCS prior to mepolizumab therapy were completely weaned off corticosteroids by the end of the study period, with no relapses, and the OCS dose taper was not associated with any clinical deterioration. This finding is in agreement with previous real-life reports, including Ueno et al. [22] and Detoraki et al. [19], who also demonstrated safe OCS dose reduction due to the OCS-sparing effect of mepolizumab.

All our patients received 300 mg of mepolizumab, and we observed improvements in BVAS, daily OCS use, and ACT scores. The studies by Wechsler et al. [17] and Ueno et al. [22] also used a 300 mg dose, reporting significant improvement in similar clinical parameters. However, some authors have reported the successful use of low-dose (100 mg) mepolizumab in the treatment of EGPA, which may further enhance the safety and cost-effectiveness of treatment. Detoraki et al. reported significant improvements in BVAS and ACT scores, and BEC and OCS use, as well as sino-nasal disease in eight patients with mepolizumab at a dose of 100 mg [19]. A case series by Vergles et al. similarly showed better asthma control and sustained relapse-free period with mepolizumab 100 mg [21]. A more recent study conducted by Bettiol et al. compared the use of 100 mg and 300 mg of mepolizumab for the treatment of EGPA in a real-life setting and found that both dose regimens were associated with adequate disease control and allowed for glucocorticoid sparing [24]. Nolasco et al. [23] also seem to show the 100 mg dose to be as effective as shown in the phase 3 trial by Wechsler et al. [17]. Three of our patients received their mepolizumab 300 mg doses every three weeks, due to inadequate disease control with the every-four-week regimen they were initially started on. The change in dosing frequency led to better disease control with no adverse events as a result of increased dose frequency. To our knowledge, there are no other reports in the literature of administering mepolizumab every three weeks to treat EGPA.

Our findings are limited by the retrospective nature of the report and the small patient number, which makes it difficult to extend the results to the overall EGPA population. Nevertheless, our findings reflect a single-center experience from the Middle East and are in line with previously published reports. Given the paucity of prospective data and only one prospective randomized controlled trial showing the efficacy of mepolizumab in EGPA [17], we feel that additional post-licensing evidence of safety and efficacy is of value to clinicians.

## Conclusions

In conclusion, we report a significant reduction in BVAS and daily OCS use after an average of 17 months of treatment with mepolizumab at a dose of 300 mg.

In our cohort, we were able to reduce the use of OCS from 12.5 mg to 2.5 mg, with 50% of patients being completely weaned off steroids. These findings are consistent with those reported previously. Further studies are necessary to identify the clinical differences between using a 300 mg dose versus a 100 mg dose for the treatment of EGPA to explore the possibility of reducing treatment costs.
